# Abnormal mitosis in reactive astrocytes

**DOI:** 10.1186/s40478-020-00919-4

**Published:** 2020-04-15

**Authors:** Alexander Sosunov, Xiaoping Wu, Robert McGovern, Charles Mikell, Guy M. McKhann, James E. Goldman

**Affiliations:** 1 Department of Neurosurgery, Columbia University, 630 W. 168th St, P&S 15-405, New York, NY 10032 USA; 2 Department of Neurosurgery, University of Minnesota, Minneapolis, MN 55455 USA; 3 Department of Neurosurgery, Stony Brook University School of Medicine, Stony Brook, NY USA; 4 Pathology & Cell Biology, Columbia University, New York, NY 10032 USA

**Keywords:** Astrocytes, Mitosis, Mitotic spindles, Polyploidy, Creutzfeldt-Peters cells

## Abstract

Although abnormal mitosis with disarranged metaphase chromosomes or many micronuclei in astrocytes (named “Alzheimer I type astrocytes” and later “Creutzfeldt-Peters cells”) have been known for nearly 100 years, the origin and mechanisms of this pathology remain elusive. In experimental brain insults in rats, we show that abnormal mitoses that are not followed by cytokinesis are typical for reactive astrocytes. The pathology originates due to the inability of the cells to form normal mitotic spindles with subsequent metaphase chromosome congression, which, in turn may be due to shape constraints aggravated by cellular enlargement and to the accumulation of large amounts of cytosolic proteins. Many astrocytes escape from arrested mitosis by producing micronuclei. These polyploid astrocytes can survive for long periods of time and enter into new cell cycles.

## Introduction

Astrocytes preserve the ability to divide in the adult central nervous system (CNS). Division is a typical feature of reactive astrogliosis, which accompanies virtually every brain injury and pathology [43, 47]. Nearly 100 years ago Creutzfeldt [12] described “giant cells” in the brains of patients with multiple sclerosis. Some of these astrocytes contained many, irregularly distributed chromosomes. Others contained multiple nuclei, some of which were small. Cavanagh and Kyu [8] described enlarged astrocytes in abnormal mitoses or cells with multiple nuclei in Wilson’s disease and other forms of hepatic encephalopathy (named “Alzheimer type I” astrocytes). Such astrocytes have been subsequently described in many other brain pathologies, including anoxic encephalopathy*,* Creutzfeldt-Jacob disease, progressive multifocal leukoencephalopathy, and brain tumors, (at present such abnormal astrocytes are usually termed “Creutzfeldt cells” [or “Creutzfeldt-Peters cells”]) [3, 14, 48].

Despite numerous studies reproducing a similar type of abnormal mitosis in many experimental conditions, the mechanisms underlying the appearance of abnormal mitosis in astrocytes in situ remain elusive [34]. Here we show that abnomal mitoses in reactive astrocytes develop as a result of the inability to perform a correct chromosome congression because of abnormalities in the mitotic spindle, correlated with changes in cell size and geometry and the large accumulation of cytosolic proteins. Escape from the arrested mitosis leads to the appearance of multinucleated, polyploid astrocytes that do not lose viability.

## Materials and methods

### Animals

Adult male rats were housed in standard cages with free access to food and water on a 12-h light/dark cycle. All procedures performed on animals were approved by Columbia University’s Institutional Animal Care and Use Committee and conducted according to institutional and federal guidelines.

### Pilocarpine induced status epilepticus

After premedication with scopolamine (5 mg/kg, i.p.) to prevent the effects of peripheral cholinergic stimulation, pilocarpine (330 mg/kg, i.p.) was administered to Sprague-Dawley rats (100–150 g) to induce seizures. Seizures were graded on the modified Racine scale [37], and only animals with grade 4–5 seizures for 2 h were used in experiments. After 2 h of continuous seizures, ketamine (80 mg/kg, i.p.) was administered to stop seizures, and a second dose (40 mg/kg, i.p.) was administered if seizures did not stop in 10 min after the first.

### Kainic acid induced status epilepticus

Kainic acid dissolved in isotonic saline (pH 7.4) was given i.p. to Sprague-Dawley rats (100–150 g) at 10 mg/kg with repeated injections of the same dose over 30 min until the appearance of grade 4–5 seizures, according to the modified Racine scale. After 2 h of continuous seizures, ketamine (80 mg/kg, i.p.) was administered to stop seizures, and a second dose (40 mg/kg, i.p.) was administered if seizures did not stop in 10 min after the first.

### Cortical stab wound model

Sprague-Dawley Rats (100–250 g) were anesthetized (ketamine 80 mg/kg, xylazine 8 mg/kg, i.p.) and placed in a stereotactic frame and the skull was exposed using sterile technique. After drilling the skull, a blunt 26-G needle (Hamilton) was inserted into the frontal cortex. 10 μl of solution (95% saline, 5% ethanol) was administered. After 96 h, animals were deeply anesthetized with an overdose of ketamine/xylazine, and perfused with 4% paraformaldehyde (PFA).

### Stroke/middle cerebral artery occlusion (MCAO)

Wistar rats (275–300 g) were subjected to transient middle cerebral artery occlusion using a method of intraluminal vascular occlusion [35]. The animals were anesthetized with halothane in a mix of 70% nitrous oxide/30% oxygen. The animals’ core temperatures were maintained at 37 °C throughout the entire procedure and for 60 min after reperfusion. The right common carotid artery, the right external carotid artery, and the right internal carotid artery were exposed and isolated. MCA occlusion was accomplished by advancing a 25 mm 4–0 nylon suture with a blunted silicone tip (outer diameter, 0.38 mm) through an incision in the external carotid artery until the suture was 18 mm past the carotid bifurcation. MCA occlusion was confirmed by transcranial measurements of cerebral blood flow via laser Doppler flowmetry (Periflux System 5000; Perimed, Inc., Järfälla, Sweden). After 120 min of ischemia, the occluding suture was removed, and reperfusion was confirmed by laser Doppler flowmetry. After 96 h, animals were deeply anesthetized with an overdose of ketamine/xylazine, and perfused with 4% PFA.

### Histology and immunohistochemistry

After perfusion brains were removed and additionally fixed in 4% PFA in PBS for 14–18 h (4^0^ C). 40 μm sections were prepared with a vibratome (Leica VT1000S) and stored in cryoprotectant solution at − 20^0^ C. Standard procedure for Nissl staining with Cresyl violet was used for routine analysis of tissue.

### Antibodies

Primary antibodies: (1) markers of astrocytes: (i) glial fibrillary acidic protein (GFAP): mouse monoclonal (1:1000, G3893, Sigma-Aldrich, St. Louis, MO), rabbit polyclonal (1:1000, Z 0334, Dako, Carpinteria, CA), phospho-GFAP (Ser8) mouse monoclonal (1:100, NBA-115, Stressgen, Ann Arbor, MI); (ii) vimentin: monoclonal (1:500, M 0725, Dako), phospho-vimentin (Ser55): mouse monoclonal (1:300, D076–3, MBL International, Woburn, MA); (iii) nestin: rabbit polyclonal (1:500, PRB-570, Covance, Emeryville, CA); astrocyte specific glutamate transporters: (iv) GLAST: monoclonal (1: 100, clone 10D4, Novocastra Lab, Newcastle upon Tyne, UK); (v) GLT1: mouse monoclonal (1:500, 611,654, BD Transduction Lab., Franklin Lakes, NJ); (vi) calcium binding protein specific for glial cells - S100: rabbit polyclonal (1:600, A 5110, Dako); (vii) alpha-B crystallin: rabbit polyclonal (1:300, SPA-223, Stressgen, Canada); (2) marker of NG2 cells: NG2 Chondroitin Sulfate Proteoglycan (1:100, AB5320, Millipore,Temecula, CA); (3) marker of microglial cells: Iba1, rabbit polyclonal (1:500, 019–19,741,Wako, Richmond, VA); (4) markers of DNA damage: phospho-gamma-H2AX (Ser139), mouse monoclonal (1:300, KAM-CC255, Stressgen, Ann Arbor, MI); (5) chromosome markers in dividing cells: (i) Ki67: mouse monoclonal (1:100, #550609, BD Pharmingen, San Jose, CA) and rabbit polyclonal (1:200, AB9260, Millipore); (ii) PCNA: mouse monoclonal (1:100, NA03, Millipore); (ii) phospho-Histone H3(Ser10): rabbit polyclonal (1:100, #9701, Cell Signaling Technology, Inc., Danvers, MA) and phospho-Histone H3 (Ser10): mouse monoclonal (1:100, #9706, Cell Signaling); (6) centrosome markers, (i) pericentrin: rabbit polyclonal (1:500, PRB-432C, Covance); (ii) gamma-tubulin: mouse monoclonal (1:100, T6557, Sigma-Aldrich) and goat polyclonal (1:100, sc-7396, Santa Cruz Technology, Inc., Santa Cruz, CA); (7) markers of microtubules: (i) alpha-tubulin: mouse monoclonal (1:500, T6074, Sigma-Aldrich); (ii) TPX2: rabbit polyclonal (1:500, Novus Biologicals, Littleton, CO); (8) marker of nuclear envelope: Lamin A/C: rabbit polyclonal (1:100, #2032, Cell Signaling): (9) markers of proteins activated in mitosis: (i) Aurora A: mouse monoclonal (1:200, A1231, Sigma-Aldrich) and rabbit polyclonal (1:100, NB100–635, Novus); (ii) Aurora B: rabbit polyclonal (1:100, A5102, Sigma-Aldrich); (iii) Bub3: mouse monoclonal (1:100, #611730, BD Biosciences) and rabbit polyclonal (1:200, NB110–40721); (iv) BubR1: mouse monoclonal (1:200, #612503, BD Biosciences, San Jose, CA) rabbit polyclonal (1:100, NB100–55254, Novus); (v) NUMA: rabbit polyclonal (1:400, NB500–174, Novus); (vi) Survivin: rabbit polyclonal (1:100, #2808, Cell Signaling); (10) Lucifer yellow: rabbit polyclonal (1:300, AB154, Millipore).

Secondary antibodies conjugated to fluorophores: anti-mouse Alexa Fluor 488, 594, and 633, anti-rabbit Alexa Fluor 488, 594, and anti-goat Alexa Fluor 488, 594, 633; all from goat or donkey (1:300, ThermoFisher Scientific, Eugene, OR).

For double- and triple-immunofluorescence, after blocking with 10% normal goat (or donkey) serum (30 min, RT), free-floating sections were incubated in a mixture of primary antibodies raised in different species overnight (4^0^ C). Alexa Fluor -conjugated secondary antibodies were used for 1 h at RT. For visualization of nuclei and chromosomes fluorescent Nissl reagent (NeuroTrace 640/660 deep-red, 1:150, ThermoFisher Scientific) and DAPI (5 μg /ml; D9542, Sigma-Aldrich) were applied with secondary antibodies.

Blocking serum, primary, and secondary antibodies were applied in 0.2% Triton X-100 in PBS. Sections for fluorescence microscopy were mounted on slides in Vectashield (Vector Laboratories, Burlingame, CA). To control for the specificity of immunostaining, primary antibodies were omitted and substituted with appropriate normal serum.

Slides were viewed using a confocal microscope (Nikon Ti Eclipse). 3D reconstructions were done from stacks of images with confocal microscope software NIS-Elements.

### BrdU administration and visualization

5-Bromo-2′-deoxyuridine (BrdU, B5002, Sigma-Aldrich, St. Louis, MO) was dissolved in sterile DPBS (10 mg/ml) and given i.p. at 80 mg/kg. At different time-points (see Results) animals were deeply anesthetized with an overdose of ketamine/xylazine, and perfused with 4% PFA. Vibratome sections were used for immunohistochemical detection of BrdU with anti-BrdU mouse monoclonal (1:330, B8434, Sigma-Aldrich) and rat monoclonal (1:100, MCA2060, AbDSerotec) antibodies after DNA denaturation: 30 min treatment with 2 M HCl (RT) followed with 20 min treatment with 0.1 M sodium borate buffer (pH 8.5) at RT.

### TUNEL

TUNEL was performed with DeadEnd™Fluorometric TUNEL system (G3250, Promega Corporation, Madison, WI) according to the manufacturer’s recommendations on vibratome sections. Regular procedures for immunohistochemical staining was performed after TUNEL.

### Electron microscopy

For regular transmission EM, animals were deeply anesthetized with a ketamine/xylazine, and perfused with 2% paraformaldehyde and 2.5% glutaraldehyde in PBS. After postfixation in 2% osmium tetroxide in 0.2 PB (2 h at 4 °C) and dehydration, pieces of tissue were embedded in Epon-Araldite (Electron Microscopy Sciences, Hatfield, PA). Ultrathin sections were cut with a Reichert Ultracut E, stained with uranyl acetate and lead citrate, and examined with a JEOL 1200 electron microscope.

### Lucifer yellow filling of astrocytes in situ

Coronal slices of rat forebrain (160–180 μm) were cut with a Vibratome (LEICA VT 1000S) in ice-cold oxygenated-modified artificial CSF (aCSF) (in mM): 125 NaCl, 2.5 KCl, 2 CaCl_2_, 1.5 MgCl_2_, 1.25 NaH_2_PO_4_, 26 NaHCO_3_, and 10 Dextrose. The slices recovered at 30 °C in a chamber for at least 1 h before electrophysiological recording. The above solution was also used for whole-cell patch-clamp recording in brain slices at 30 °C. The bath solution was applied at a flow rate of 1.5 ml/min using the VC-6 perfusion valve control system (Warner Instruments) with the TC-344B temperature controller. Cells were visualized under a LEICA DMLFS microscope with a 63× water-immersion lens. The intracellular solution in the patch pipettes contained the following (in mM): 140 KCl, 1 MgCl_2_, 10 EGTA, 10 HEPES, 3 MgATP, 0.3 Na_2_ATP, pH 7.3 with KOH. Pipette resistance was ∼3–5 MΩ. Cell capacitance and series resistance were measured using the software MultiClamp 700A Commander Ver. 1.1.2.27 and pClamp 8 (Axon Instrument, Molecular Devices). Cells were initially identified morphologically based on the sizes and shapes of their somas and the architecture of their processes. For analysis of cell morphology and gap junction coupling with the surrounding cells, Lucifer yellow (LY, Sigma-Aldrich) was added to the intracellular solution (final concentration 0.1%) and filtered through a 0.2 μm PTFE filter. After the experiment, slices were fixed in 4% paraformaldehyde in PBS overnight at 4 °C. Slices were immunostained, and observed under a confocal microscope, as above.

### Quantitative analysis

The numbers of mitotic astrocytes visualized with Ki67 or BrdU were counted in the images (merged from stacks of 6 adjacent images with 1024 × 1024 pixel resolution in an observed area of 295 × 295 μm, captured with a confocal microscope at a distance of 0.5 μm from each other) obtained from neocortices in coronal sections (10 images from each section, 5 sections per animal).

Levels of GFAP immunofluorescence were evaluated in the images obtained as described above from coronal brain sections stained for GFAP and Ki67. Images were transferred to Image J 1.46r (public domain), grayscaled and quantified based on the optical density (OD).

### Statistical analysis

Data are expressed as mean ± SEM. Continuous parameters were analyzed with Student’s *t*-test and one-way Anova. *p* < 0.05 was considered significant.

## Results

### The appearance of arrested (C-like) mitoses and multinucleation are typical features of reactive astrogliosis

**Fig. 1:**
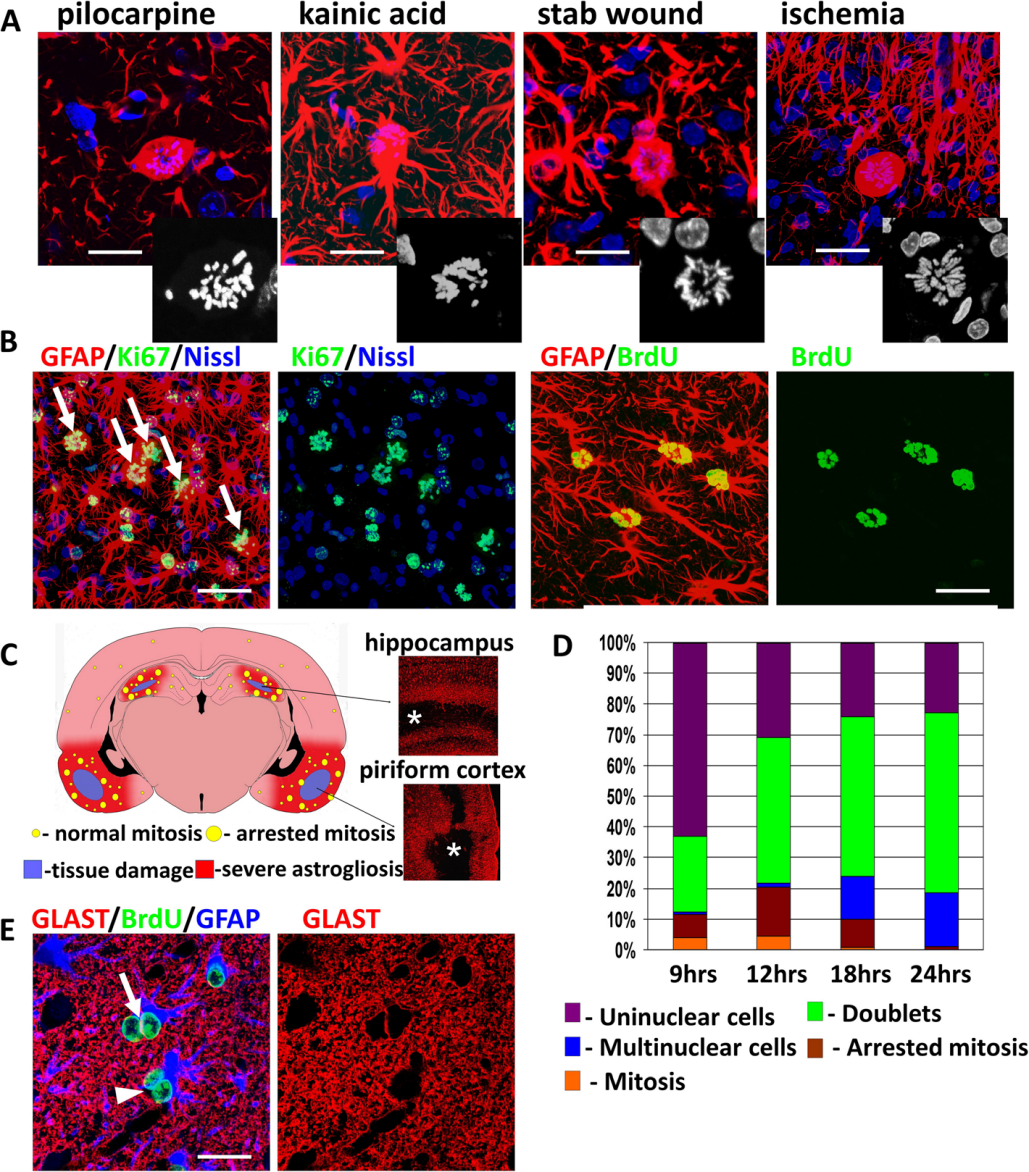
Abnormal mitoses are common features of reactive astrogliosis. **a** Examples of astrocytes arrested in metaphase in excitotoxic (pilocarpine [neocortex] and kainic acid [hippocampus, CA1]), mechanical (stab wound [neocortex]), and ischemic [neocortex] brain damage. Note enlargement of astrocyte cell bodies, reduction of the numbers of main branches, and abnormal positions of metaphase chromosomes distributed over the entire volume of the cell bodies (black and white insets of DAPI stains in lower right segments of each panel). Immunostaining for GFAP, counterstaining with DAPI. Confocal microscopy. **b** Arrested mitoses in astrocytes (arrows, left 2 images) and astrocytes with many small nuclei (right 2 images). One day after BrdU (80 mg/kg) administration on day 3 after pilocarpine administration, piriform cortex. Confocal microscopy. **c** Schematic presentation of the most damaged brain areas after pilocarpine administration and the locations of arrested mitoses. Note in the right panels (immunostaining for GFAP) that areas of tissue damage (asterisks) in hippocampus and piriform cortex are devoid of GFAP immunostaining. **d** Time scale of the dynamics of astrocyte cycling after one BrdU dose (80 mg/kg, i.p.) given on day 3 after pilocarpine administration. Sections were examined 9–24 h after BrdU administration. **e** Example of ‘doublets’ when daughter astrocytes do not migrate away from each other after mitosis (arrow, note that neighboring nuclei are separated by plasma membranes visualized with GLAST) and when a mitosis was not followed by cytokinesis, generating a binucleated astrocyte (arrowhead, note that the nuclei are not separated by plasma membranes). Scale bars:  a = 10 μm, b = 45 μm, e = 20 μm

We found many abnormal mitoses in astrocytes in excitotoxic (kainic acid and pilocarpine-induced), ischemic, and traumatic (stab wound) models of brain injury in rats (Fig.1a). These cells were usually located in the vicinity of the damaged tissue in the area of severe astrogliosis. The model with pilocarpine administration was especially suitable for analysis of mitotic abnormalities in astrocytes because it gave standard and reproducible results with many abnormal mitoses. Two brain areas regularly showed severe neuronal damage and an abundance of astrocyte mitoses: the piriform and entorhinal cortices and the hippocampus (Fig. 1c). The maximal mitotic activity of astrocytes was on day 4 after pilocarpine administration, after which mitoses gradually decreased and were minimal after day 7. On day 4 after seizure induction, about 45% of astrocytes near the damaged areas were Ki67+. In single coronal sections we found 89 ± 32 mitotic astrocytes (in M phase); nearly 20% of them showed abnormal C-like-mitoses or were multinucleated (Fig. 1b). Multinucleated cells with many micronuclei (restitution nuclei) were considered a result of cellular escape from arrested mitosis (Fig.1b). To examine the kinetic characteristics of astrocyte cell cycling further, one dose of BrdU (80 mg/kg) was given on day 3 after pilocarpine administration and animals were sacrificed at different times thereafter, up to 24 h (Fig. 1d). By 24 h, the majority of BrdU+ astrocytes were found in doublets (paired astrocytes) seen as two BrdU+ cells in close apposition to each other. Analyses with plasma membrane markers (astrocyte-specific glutamate transporters GLT-1 and/or GLAST) showed that ~ 15% of doublets (45 of 279 doublets examined) were cells with two nuclei, indicating an absence of cytokinesis after mitosis (Fig. 1e). We never observed abnormal mitoses in NG2+ oligodendrocyte precursor cells or microglia. To the best of our knowledge, there are no reports of the appearance of abnormal or arrested mitoses in these cells.

### DNA damage does not accompany arrested C-like-mitoses

**Fig. 2:**
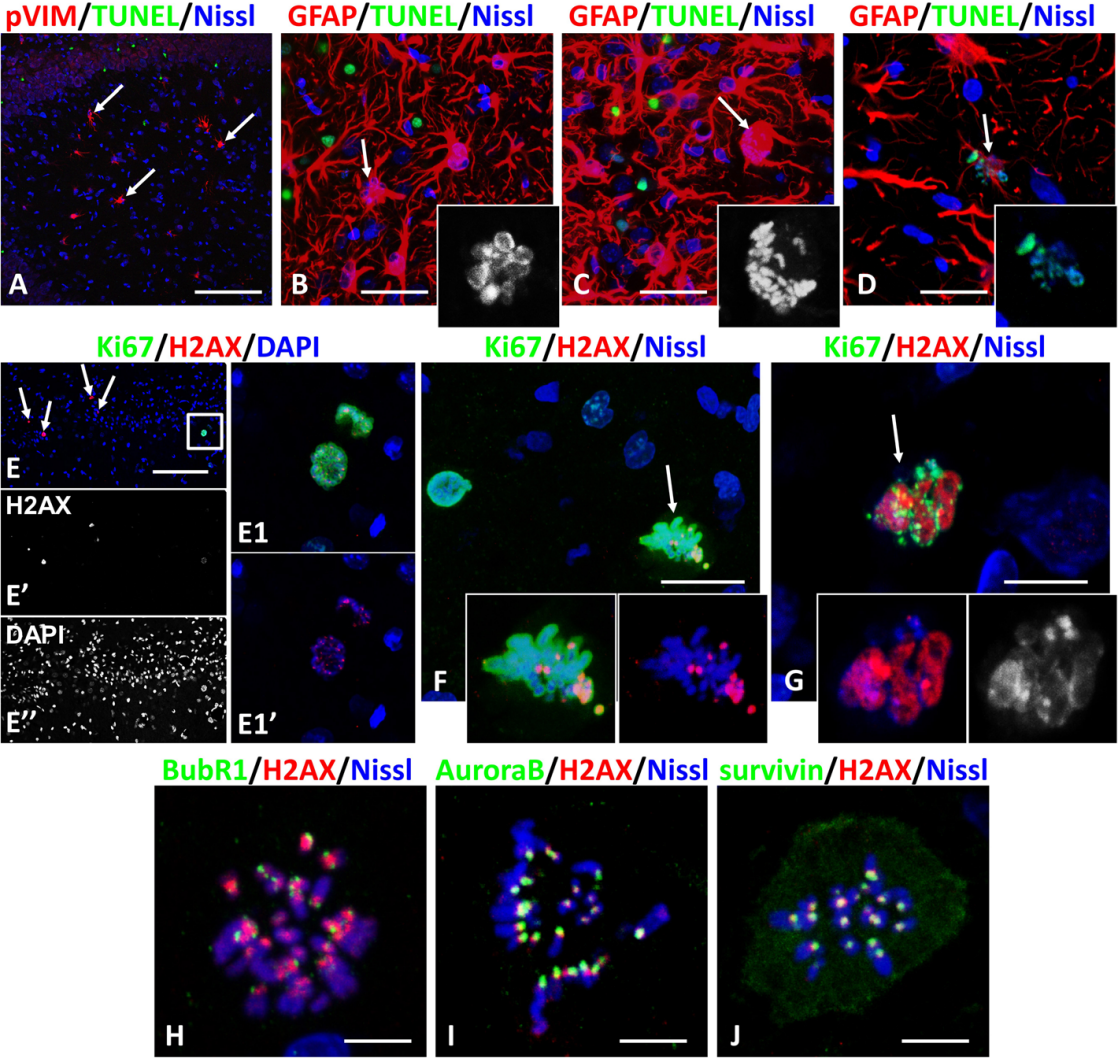
DNA damage does not accompany arrested C-like-mitoses. **a-d** DNA fragmentation detected with TUNEL is not typical for astrocytes arrested in mitosis. **a** CA1 hippocampal subfield with many mitotic astrocytes (identified with phospho-vimentin (pVIM), some of the astrocytes arrested in metaphase are indicated with arrows) that are not positive for TUNEL. Note that the TUNEL+ green nuclei are located in the pyramidal layer and belong to neurons. **b, c** Piriform cortex, astrocyte with many small nuclei (arrow in **b**) and astrocyte arrested in metaphase (arrow in **c**) do not show TUNEL staining. Note that many nuclei are TUNEL+, but are not astrocytes. **d** TUNEL+ staining of misaligned metaphase chromosomes indicating DNA fragmentation in this astrocyte (arrow). Hippocampus, CA1 subfield. Insets in lower right parts in (**b**, **c**, and **d**) show enlarged nuclei and metaphase chromosomes in the cells marked with arrows in (**b**, **c**, **d**) respectively. **e**-**g** Immunoreactivity for phospho-gamma-H2AX (H2AX) is present only in small numbers of mitotic astrocytes. **e** H2AX+ nuclei (arrows) in the CA1 layer of pyramidal neurons. Note in E that many neurons displayed shrunken, condensed nuclei indicating apoptotic changes. e1, e1’) enlarged boxed area in E shows mitoses. Note that positive signals for H2AX are localized in small, focal areas of chromosomes. **f**, **g** Immunoreactivity for H2AX is present in some metaphase chromosomes (f, arrow) and in micronuclei (**g**, arrow) indicating DNA breaks. **h**, **i**, **j** Colocalization of H2AX with markers of kinetochores: BubR1 (**i**), Aurora B (**j**), and survivin (**k**). **f**-**j** – piriform cortex. All images were obtained with confocal microscope 4 days after pilocarpine administration. Scale bars:  a = 150 μm,  b-d = 35 μm,  e = 125 μm,  f,g = 8 μm, h-j = 3 μm

To determine whether abnormal mitoses might be due to DNA damage, we used TUNEL (as a marker of DNA cleavage) and phospho-gamma-H2AX (H2AX, an early marker of DNA double-strand breaks) to evaluate DNA damage in astrocytes. Only one TUNEL+ astrocyte in C-mitosis was found out of 376 cells detected in C-mitosis and with many small nuclei on days 3–6 after induced seizures (Fig. 2a-d). A typical feature of this model is the extensive neuronal death. Labeled nuclei were present in neurons near the areas with damage and served as a positive control (Fig. 2e). In contrast, high levels of H2AX immunostaining were only rarely found in astrocytes (4 of 214), in some parts of metaphase chromosomes, and in some micronuclei. These were considered to be loci of DNA damage (Fig.2f,g). Immunostaining for H2AX in kinetochore/centromere chromosome loci was observed in all mitotic astrocytes (Fig. 2e1,h-j), similar to that which occurs in other types of dividing cells [4, 16]. Thus, DNA damage is not a major cause of arrested mitoses in reactive astrocytes. We also conclude that arrested mitoses in these astrocytes do not cause significant DNA damage, which can accompany prolonged mitotic arrest [42].

### Retraction of main branches with loss of miniature processes and loss of intracellular coupling is a prominent feature of mitotically arrested astrocytes

**Fig. 3:**
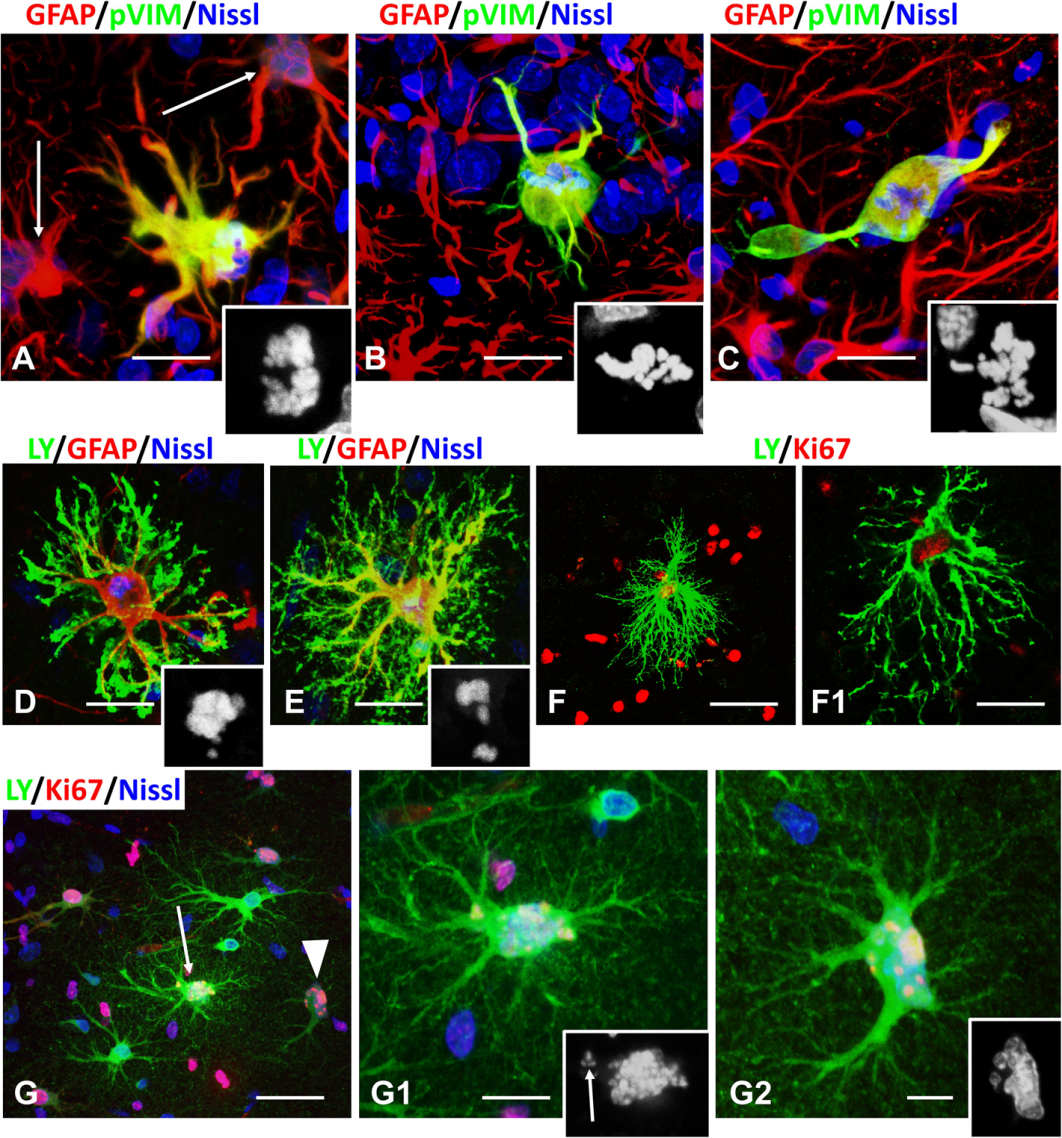
Changes in astrocyte morphology in mitotic arrest. **a**-**c**. **a** Astrocyte with enlarged cell body and many primary processes. Note that two neighboring astrocytes (arrows) with micronuclei reveal minimal immunoreactivity for phospho-vimentin (pVIM) indicating a primarily non-phosphorylated state after mitotic slippage. **b** Astrocyte with a ball-like cell body and only a few primary processes. **c** Astrocyte showing a significant reduction of primary processes. Insets show misaligned chromosomes with DAPI. **d**-**f** Mitotic astrocytes filled with Lucifer yellow (LY) reveal a reduction in numbers of miniature distal processes. Insets in (**d** and **e**) show disarranged metaphase chromosomes. f1 is a single optical slice of an enlargement of the astrocyte in (**f**). Note that the astrocytes in (**d-f**) are not coupled with neighboring astrocytes. **g** An astrocyte filled with LY (arrow, enlarged in g1) contains misaligned chromosomes (enlarged in inset in g1), but retains a bushy-like appearance and is coupled to neighboring astrocytes, one of which (arrowhead in g, enlarged in g2) has several micronuclei, indicating slippage from arrested mitosis. Insets show metaphase chromosomes (g1, arrow indicates misaligned chromosomes) and micronuclei (g2). All images were obtained with confocal microscope 4 days after pilocarpine administration. Scale bars: a-c = 10 μm,  d,e = 15 μm,  f = 35 μm, g = 35 μm, g1, g2 = 10 μm

Many astrocytes arrested in M-phase preserved their main branches and their stellate shapes (~ 60%, 240 of 392 astrocytes arrested in M phase), whereas others showed ball-like cell bodies with only a few main processes, as judged by GFAP and vimentin immunostaining (Figs. 3a,b,c,). To look further at astrocyte shapes, we filled cells with Lucifer yellow during electrophysiological recordings. This revealed astrocytes in which the main cellular processes were devoid of the small leaf-like processes that typically enwrap synapses and are responsible for the bushy appearance of normal astrocytes (Fig. 3d-f,f1). Lucifer yellow injections also allowed us to examine coupling between adjacent astrocytes. In parallel with the disappearance of miniature processes, astrocytes lost their coupling (compare astrocytes in Fig. 3d-f to astrocyte in Fig. 3g that is coupled to 2 neighboring cells). Only a minority of astrocytes in arrested mitosis preserved the normal, bushy appearance and coupling (Fig. 3g).

### Reactive astrocytes alter the correct positioning of the mitotic spindle and chromosome congression

**Fig. 4:**
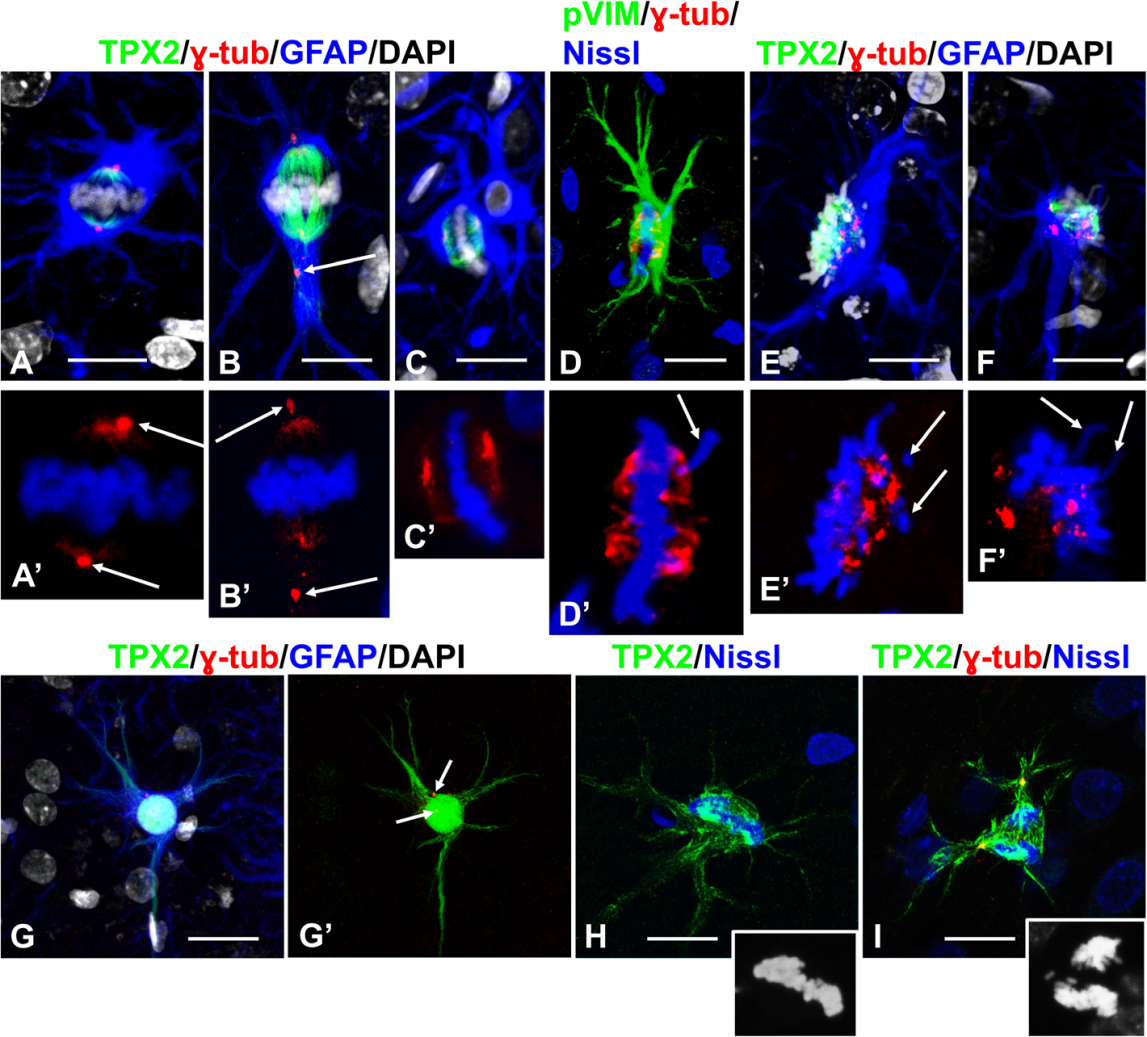
Orientation of mitotic spindles (upper panel) and TPX2 immunoreactivity in mitotic astrocytes (lower panel). **a**, **b** Mitotic spindles are oriented along the long cellular axes. Note that: 1) immunoreactivity for gamma-tubulin (γ-tub) is localized in centrosomes (arrows in a’ and b′) and at the poles of the spindles; 2) in (**b**) that one centrosome is displaced into a proximal part of a process (arrow in **b**) and that this centrosome co-localizes with TPX2 in the process; 3) areas occupied with spindles are devoid of GFAP immunoreactivity. For an animated 3D reconstruction of the spindles see Additional files 4a and 4b. **c**, **d** The mitotic spindle is oriented perpendicular to the long cellular axis. Note the widening of the spindle poles, the appearance of additional foci of gamma-tubulin reactivity, and lagging chromosome (arrow) in c′d’). For an animated 3D reconstruction of the spindle in (**c**) see Additional file 4C. **e, f** Metaphase chromosomes located at the periphery of cell body. Note several foci of gamma-tubulin immunoreactivity and lagging chromosomes (arrows) in (e’,f’). For an animated 3D reconstruction of the spindles see Additional files 4E and 4F. **g**-**i** TPX2 immunoreactivity in mitotic astrocytes is present in cell body and processes indicating penetration of astral microtubules in processes. **g** Prophase. g’ shows two channels TPX2 and γ-tubulin. Centrosomes are indicated with arrows. **h** Metaphase. **i** Anaphase. Lower right insets in (**h** and **i**) show chromosomes. All images were obtained with confocal microscope. Scale bars: a-f = 10 μm,  g-i = 15 μm

**Fig. 5:**
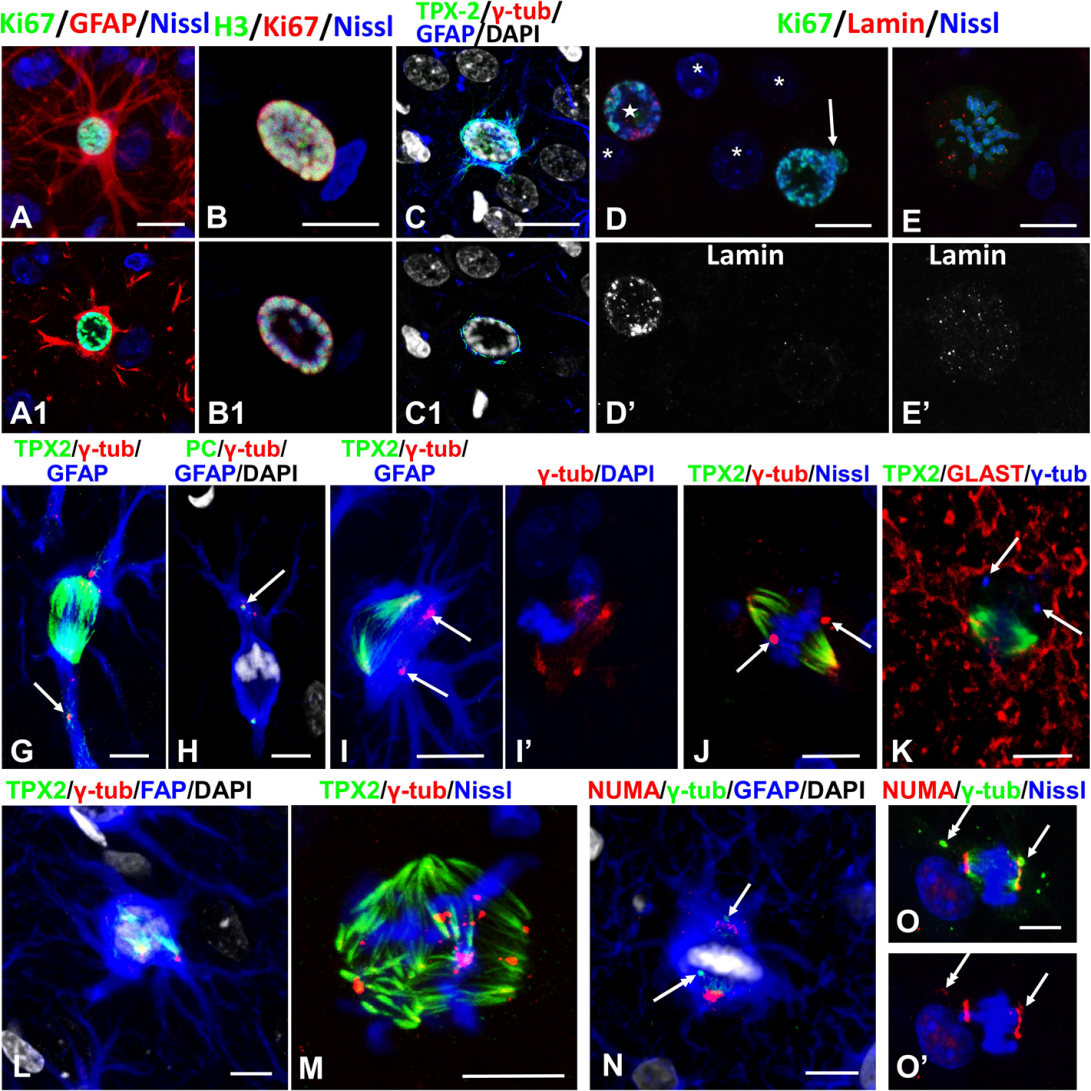
Aggregation of condensed chromosomes near nuclear envelope at the onset of nuclear envelope breakdown (NEB) (upper panel) and displacement of centrosomes from spindle poles (lower panel). **a**-**c** Condensed chromosomes are localized near the nuclear envelope. **a**, **b**, **c** –merged confocal images including the entire volume of the nucleus,a1,b1,c1 – single optical slices from central part of each nucleus. Note in cthat microtubules visualized with TPX2 encircle the nucleus and also appear in the processes. For an animated 3D reconstruction of (**a**) see Additional file 5A. **d** Disappearance of lamin A/C (lamin) immunoreactivity in the nucleus with chromosomes aggregated near the nuclear envelope. Note that some parts of chromosomes are located outside of the envelope profile (arrow), indicating NEB. Note also that nuclei of neighboring cells (probably neurons) do not show lamin A/C immunoreactivity (asterisks), whereas the nucleus of the astrocyte marked with a star displays lamin A/C immunostaining. **e** In arrested mitosis lamin A/C immunoreactivity has a punctate appearance distributed over the cytoplasm of the cell body. (**g,h**) Displacement of centrosomes (identified with gamma-tubulin [γ-tub] and pericentrin [PC], arrows) from spindle poles into the proximal parts of processes. For an animated 3D reconstruction of (**g**) see Additional file 5G. **i**-**k** Displacement of centrosomes (arrows) away from spindle poles. i′ shows two channels γ-tub and DAPI from three shown in i. (**j**) Note that centrosomes (arrows) are aside from poles. **k** GLAST immunostaining delineates the cell body of the astrocyte. For an animated 3D reconstruction of (**i, j**) see Additional files 5 I, J. **l**, **m** Several pole-like foci produced by kinetochore microtubules. Note multiple foci of gamma-tubulin immunoreactivity in (**m**). For an animated 3D reconstruction of (**l**, **m**) see Additional files 5 L, M. **n**, **o** NUMA immunoreactivity was predominantly located in the areas of kinetochore microtubule convergence in the spindle pole-like structures. Note that centrosomes (arrow and double headed arrow) are not associated with high immunoreactivity for NUMA and that centrosomes (double headed arrows) are displaced from spindle poles. In o′, NUMA and Nissl are shown without γ-tub. For an animated 3D reconstruction of (**n**) see Additional file 5 N. All images were obtained with confocal microscope. Scale bars:  a,c = 15 μm, b,d,e = 7 μm, g-n = 10 μm, o = 6 μm

We examined the orientation of spindles in reactive astrocytes. To do this we used immunoreactivity to TPX2, a microtubule-associated protein required for microtubule nucleation, as a marker for growing microtubules [1] [53]. TPX2 immunoreactivity was clearly observed throughout most of the depth of the slices (in contrast to α or β tubulin immunolabeling that was observed only superficially), allowing us to analyze entire mitotic spindles. TPX2 immunoreactivity was also observed in the processes of mitotic astrocytes in prophase, metaphase and anaphase (Fig. 4g-i) and around the nucleus in prophase (Fig. 4g) especially when condensed chromosomes were gathered near the nuclear envelope (Figs. 5c, 7a).

In many astrocytes the spindles displayed a normal appearance and orientation (Figs. 4a,b; 5g; Additional movie files show this in more detail [See Additional files 4A,B; Additional file 5G]), whereas in other cells in M phase the chromosomes were located at the periphery of the cell body and the orientation of the spindles varied from this normal appearance (~ 72%, 156 of 215 cells selected randomly and analyzed for the location of metaphase chromosomes). Some spindle poles appeared orthogonal to the long axis of the cell, which led to an expansion of pole areas and the appearance of additional foci of gamma-tubulin immunoreactivity (Fig. 4c,d; Additional movie files show this in more detail. [see Additional files 4 C,D]). Many spindles were misshapen with several foci of gamma-tubulin immunoreactivity and misaligned chromosomes located beside the main group (Fig. 4e,f; Additional movie files show this in more detail. [see Additional files 4 E,F]).

The position of condensed chromosomes at the time of nuclear envelope breakdown is an important factor for the binding of centromeres with kinetochore microtubules and chromosome segregation into the metaphase plate. The majority of condensed chromosomes in large nuclei were located near the nuclear envelope, leaving the central part of the nucleus free. Such localization was especially obvious in single optical slices taken from the central part of the nucleus (Fig. 5, compare a,b,c and a1,b1,c1, respectively; An additional movie file shows this in more detail. [see Additional file 5A]). Note that both the BUB3 and BUBR1components of the spindle assembly checkpoint were identified in chromosomes at this position (Fig. 7a). The peripheral position of chromosomes was preserved even at the onset of nuclear envelope breakdown, which we defined as a disruption of the even outlines of the nuclei by chromosomes protruding outside the nuclear profile (Fig. 5d). In such nuclei, lamin A/C immunoreactivity, which was used as a marker of the nuclear envelope, was diminished or appeared as isolated, small foci all over the nucleus (Fig. 5d). Small, puncta-like lamin immunoreactivity in the cytoplasm was typical for arrested metaphase and probably represent small fragments of the nuclear envelope (Fig. 5e). We do not assume that such spatial organization of chromosomes at the time of nuclear envelope breakdown is typical for mitotic arrest, but in combination with other factors it may impede chromosome congression.

### Centrosomes are displaced from spindle poles

When spindle poles were located near the beginning of main astrocyte processes, centrosomes (identified with pericentrin and/or gamma tubulin) were often displaced into the proximal parts of the processes at distances up to 11 μm from the spindle poles (defined as a point at which the kinetochore microtubules converged) (Figs. 4b,i,g,h5; Additional movie files show this in more detail. [see Additional files 4B, 5G]). We observed such displacement of centrosomes in ~ 30% of astrocytes (78 of 235). When the orientation of the spindles was perpendicular to the long axis of the perikaryon or when chromosomes were located in the periphery of the cell bodies, centrosomes were often found off to the side of spindle poles (Fig. 5i-k; Additional movie files show this in more detail. [see Additional files 5 I, J,L]). Another prominent abnormality was the appearance of additional spindle pole(s), when converging kinetochore microtubules produced several cone-like structures in addition to two main poles (Fig. 5l,m; Additional movie files show this in more detail. [see Additional files 5 L,M]).

We then examined the distribution of the nuclear mitotic apparatus protein, NUMA, a microtubule-binding protein that regulates the formation and maintenance of spindle poles, tethering microtubules at the poles during mitosis, and the alignment and segregation of chromosomes [44]. In abnormal mitoses, NUMA did not co-localize with centrosomes when they were displaced from the spindle poles (Fig. 5n,o; Additional movie file shows this in more detail. [see Additional file 5 N]), although NUMA appeared to be co-localized with microtubules at spindle poles. Thus, NUMA does not regulate the centrosome localization to spindle poles in abnormal mitoses.

### Abnormal loci of microtubule nucleation are typical for abnormal mitotic spindles

**Fig. 6:**
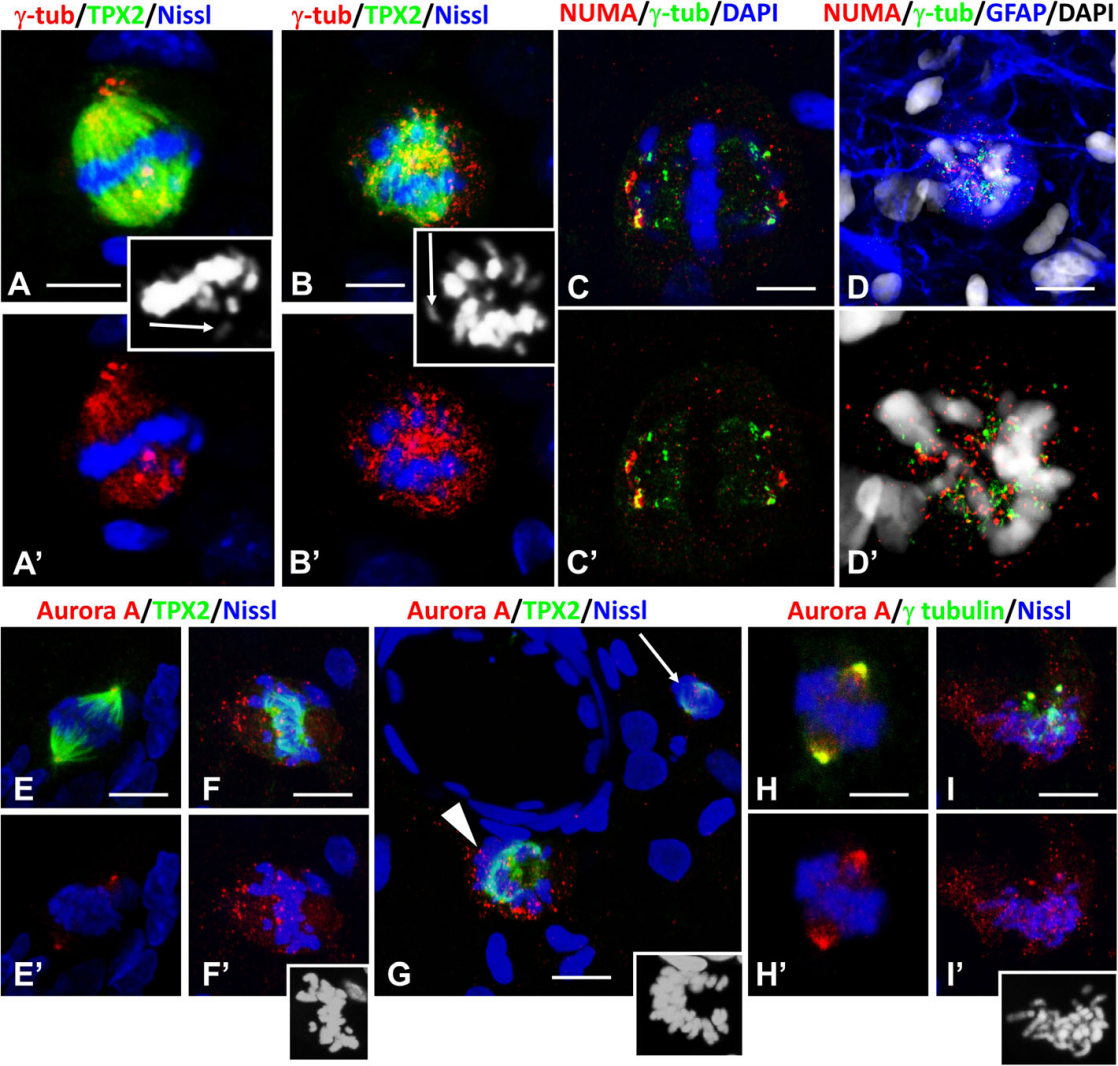
Appearance of additional gamma-tubulin+ foci of microtubule nucleation (upper panel) and immunoreactivity for Aurora A in arrested mitoses (lower panel). **a**, **b** Several foci with gamma-tubulin (γ-tub) immunoreactivity in the vicinity of metaphase chromosomes, some of which are misaligned (arrows in the insets). a’ and b’ – split (**a** and **b**) images, respectively. Insets show position of chromosomes. For an animation of 3D reconstruction of (**a**, **b**) see Additional files 6A,B. **c**, **d** Foci of NUMA immunoreactivity are distributed all over the area occupied by metaphase chromosomes, many of which are misaligned and only partly colocalize with gamma-tubulin. c and c’ – split (**c** and **d**) images, respectively. For an animation of 3D reconstruction of (**c**, **d**) see Additional files 6C, D. **e**-**i** Immunoreactivity for Aurora A in normal mitoses (**e**, **h**) and abnormal mitoses with misaligned chromosomes (**f**, **g**, **i**). Note that in normal mitosis Aurora A localizes predominantly in the centrosomes, whereas in mitoses with misaligned chromosomes, small foci of Aurora A immunoreactivity are distributed over a large volume around the chromosomes. Note in G the difference between arrested mitosis (arrowhead) and normal mitosis (arrow). Lower right insets show positions of the chromosomes. All images were obtained with confocal microscope. Scale bars: a,b,d,f,h,i = 5 μm, c = 3 μm, = 10 μm, g = 8 μm

The modern paradigm of mitotic spindle formation includes two main mechanisms: a centrosome-based and a chromosome-based center of microtubule nucleation, the latter involving a small GTPase Ran gradient in the vicinity of kinetochores [52]. In centrosomes, gamma-tubulin is a major component of the microtubule assembly complex [31]. In normal astrocyte mitosis, gamma-tubulin is localized mainly in centrosomes (Fig. 4a). However, when chromosomes were misaligned in reactive astrocytes, additional gamma-tubulin+ foci appeared in the vicinity of spindle poles and chromosomes (Figs. 4d,e,f; 6 a,b; Additional movie files show this in more detail. [see Additional files 6 A,B]). NUMA was also abnormally distributed, with the appearance of many small immunoreactive foci and only partly colocalized with gamma-tubulin (Fig. 6c,d; Additional movie files show this in more detail. [see Additional files 6 C,D]).

Aurora A kinase is critical for spindle maturation and for centrosome separation and maturation (e.g. recruiting gamma tubulin) [6, 18]. In normal astrocyte mitoses Aurora A was predominantly concentrated in the centrosomes (Fig. 6e,h), whereas in abnormal mitoses Aurora A immunoreactivity appeared as small foci spread over a large volume of cytoplasm around the chromosomes (Fig. 6f,g,i), and only some Aurora A+ foci were colocalized with gamma tubulin (Fig. 6i). Thus, several molecules that are components of a normal centrosomal microtubule assembly complex are no longer localized properly.

### Mitoses arrested in metaphase display features of unsatisfied spindle assembly checkpoint (SAC)

**Fig. 7:**
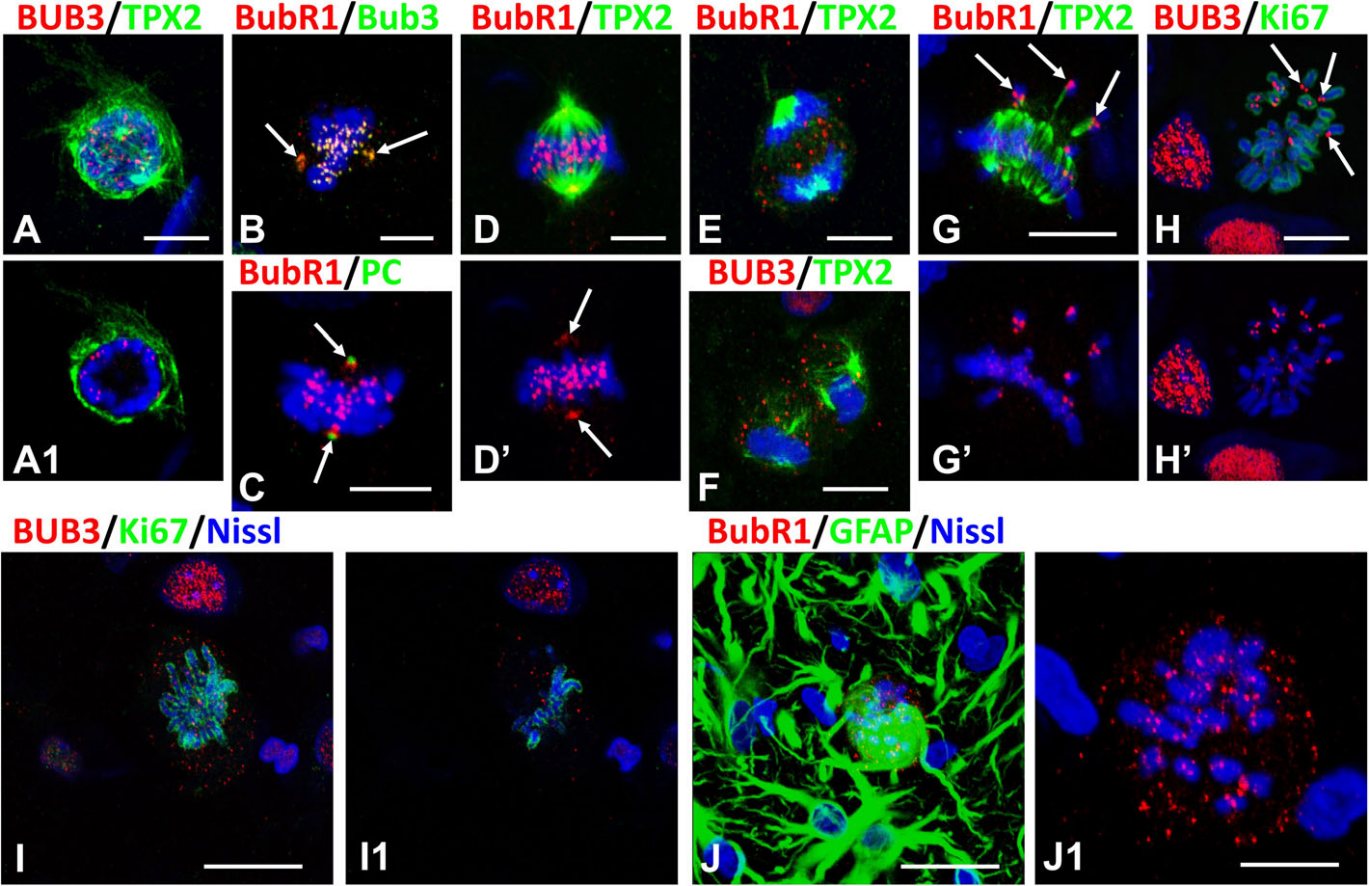
Location of BubR1 and BUB3 in mitotic astrocytes. **a**-**f** Normal mitoses. In prophase (**a**) and metaphase (**b**-**d**) BubR1 and BUB3 are found in kinetochores and centrosomes (arrows). a1 – single optical slice from stack of images in (**a**). Note the abundance of microtubules visualized with TPX2 covering the nucleus. **e**, **f** In anaphase BubR1 and BUB3 are distributed between segregated daughter chromosomes. **g**, **h** Immunoreactivities for BubR1 and BUB3 predominated in kinetochores of lagging chromosomes (arrows). **i**, **j** In arrested mitoses many foci of BubR1 and BUB3 immunoreactivity are not associated with chromosomes. i1 and j1 – single optical slices from stacks in (**i** and **j**), respectively. j1 – enlarged area in j with chromosomes, GFAP immunostaining is not shown. All images were obtained with confocal microscope. Scalebars: a-h = 5 μm,i = 8 μm,  j = 20 μm, j1 = 8 μm

SAC prevents the onset of anaphase before all chromosomes are properly attached to kinetochore microtubules and therefore can be segregated correctly [19, 30, 38]. We examined immunoreactivity for the SAC kinase, BubR1 and the SAC protein, BUB3. In a normal astrocyte mitosis these proteins first appeared in prophase in condensed chromosomes located near the nuclear envelope (Fig. 7a, only BUB3 is shown here) and in metaphase they were found in kinetochores and centrosomes, their normal localizations (Fig. 7b-d). In anaphase immunoreactivity for BubR1 and BUB3 was observed as puncta distributed between segregating daughter chromosomes (Fig. 7e,f).

In arrested mitoses the distribution of BubR1 and BUB3 was altered. Their immunoreactivity predominated in kinetochores of chromosomes that were located apart from the main group of chromosomes (Fig. 7g,h). When chromosomes were chaotically spread over a large volume, many small foci of immunoreactivity were distributed in the cytoplasm around the chromosomes (Fig. 7i, j). These SAC proteins, therefore, only partly follow chromosome kinetochores when chromosomes are not aligned properly in the mitotic figure, and are found outside of centromeres. We do not know whether SAC protein mis-localization follows mis-alignment of chromosomes, or contributes to it.

**Fig. 8:**
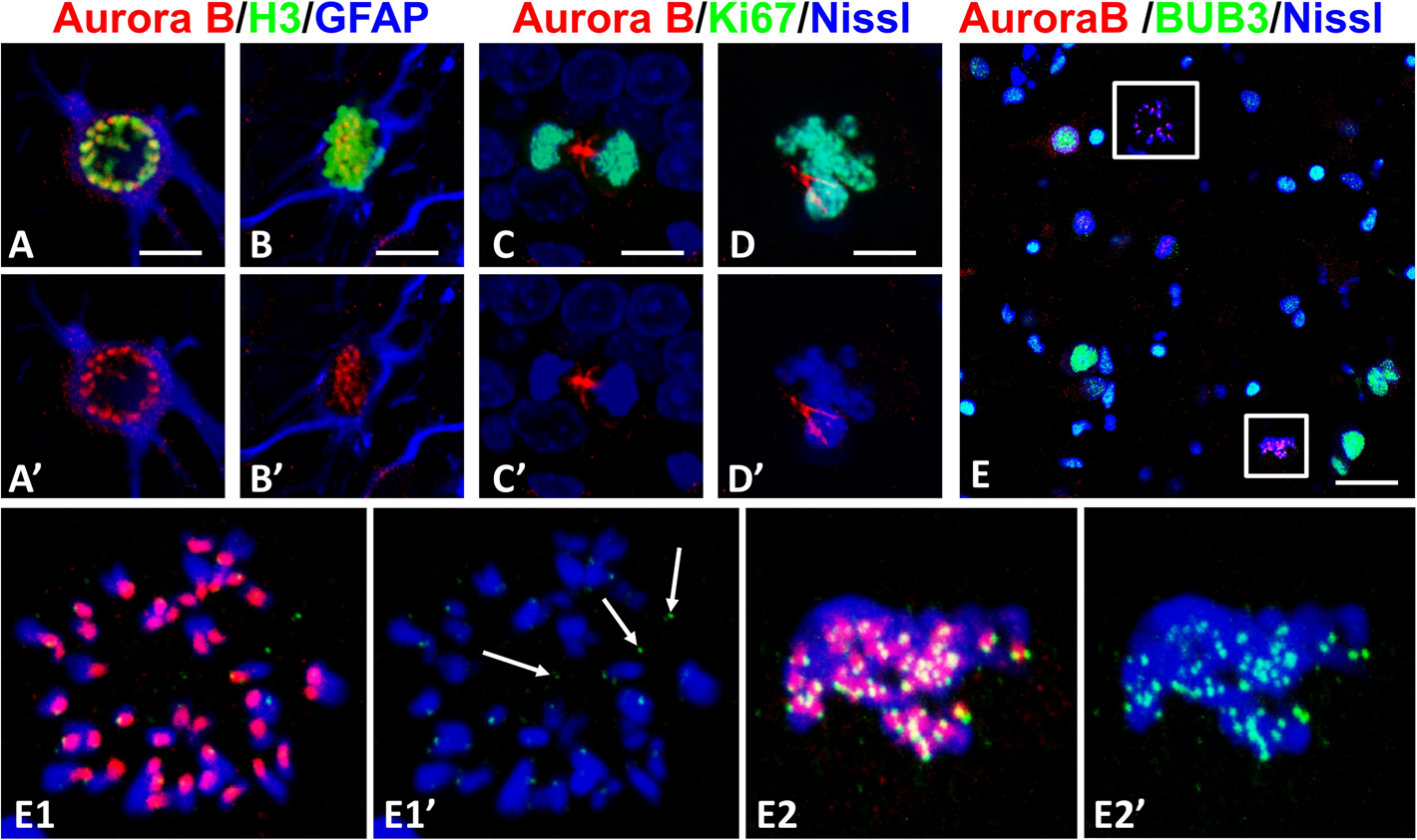
Aurora B in mitotic astrocytes. **a**-**c** Aurora B immunoreactivity in normal mitoses, in prophase (**a**), metaphase (**b**), and telophase (**c**). Note that in prophase and metaphase Aurora B is found in focal areas in chromosomes (kinetochores). **d** Typical pattern of Aurora B immunoreactivity in multinucleated astrocytes. **e** Double immunostaining for Aurora B and BUB3. e1 – enlarged upper boxed area with arrested mitosis. Note that BUB3 immunoreactivity is minimal, whereas Aurora B is preserved in kinetochores. e2- enlarged lower boxed area with chromosomes congressed in metaphase plate. Note that both Aurora B and BUB3 are colocalized at kinetochores in congressed chromosomes (e2) but in arrested mitosis (e1) where BUB3 (arrows) but not Aurora B is found not connected with chromosomes. All images were obtained with confocal microscope. Scale bars:  a-d = 15 μm, e = 75 μm

Aurora B kinase is responsible for the attachment of kinetochore microtubules to chromosomes and fixes incorrect connections between chromosomes and microtubules [26, 28]. It belongs to the chromosomal passenger complex of proteins that have a typical distribution during mitosis (Fig. 8a-d). In arrested mitoses Aurora B was found in kinetochore areas but not always co-localized with BuB3 (Fig. 8e; compare Fig. 8e1 showing typical arrested mitosis and Fig. 8e2 showing chromosomes during congression, when BuB3 levels appear higher it is associated with Aurora B and chromosomes).

### Cytoplasmic proteins may interfere with the formation of spindles and chromosome congression

**Fig. 9:**
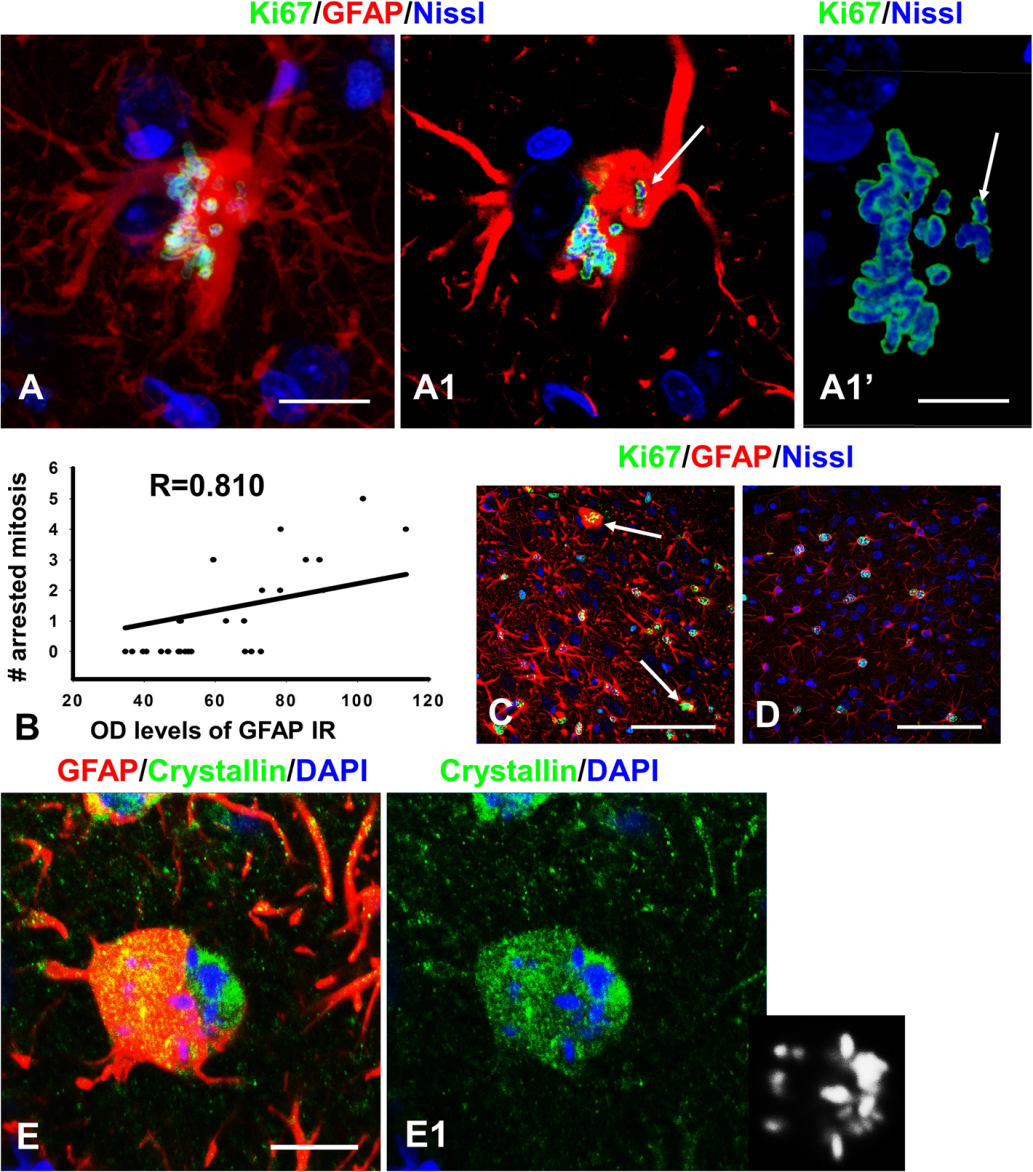
Levels of GFAP and alphaB-crystallin immunoreactivities correlate with the appearance of abnormal mitoses. **a** Lagging chromosomes (arrow) are separated from the main group by cytoplasm with high levels of GFAP immunoreactivity. a1 – single optical slice. a1’- enlarged image of chromosomes in a1. For an animated 3D reconstruction of (**a**) see Additional file 9A. **b** Significant positive correlation between levels of GFAP immunoreactivity and numbers of arrested mitoses. Regression analysis, R = 0.810. **c**, **d** In areas of severe astrogliosis (**c**) there are astrocytes with arrested mitosis (arrows); in mild astrogliosis (**d**) at a distance from the damaged area arrested mitosis are not observed. **e**) alphaB-crystallin (Crystallin) is located around chromosomes in arrested mitosis. Right lower inset shows position of chromosomes. All images were obtained with confocal microscope. Scale bars: a = 20 μm,  c,d = 80 μm,  = 18 μm

After the breakdown of the nuclear envelope, the cytoplasm gains access to the peri-chromosomal space and may interfere with chromosome congression. In normal mitosis, the area occupied by the mitotic spindle and chromosomes is free from many cytoplasmic proteins, for example, GFAP, the major intermediate filament of astrocytes (Fig. 4a,b). However, in many astrocytes with abnormal mitosis the lagging chromosomes and centrosomes were often located in areas with high levels of GFAP immunoreactivity (GFAP-IR) (Fig. 9a). Thus, an abundance of proteins may be a structural obstacle for correct spindle formation and chromosome congression (Fig. 9a; Additional movie file shows this in more detail. [see Additional file 9A]). Quantitative correlation between the numbers of abnormal mitosis (arrested C-like mitoses and multinuclear cells) and the level of GFAP-IR revealed a high, positive correlation between these two variables (linear regression, R = 0.81) (Fig. 9b). We also compared areas of severe to those with mild astrogliosis, measured by the relative levels of GFAP-IR, (Fig. 9c, d) and found a significant difference in the numbers of arrested mitoses (7.3 ± 1.5 per 1mm^2^ in severe vs 0.5 ± 0.3 per 1mm^2^ in mild astrogliosis, *P* < 0.001).

**Fig. 10:**
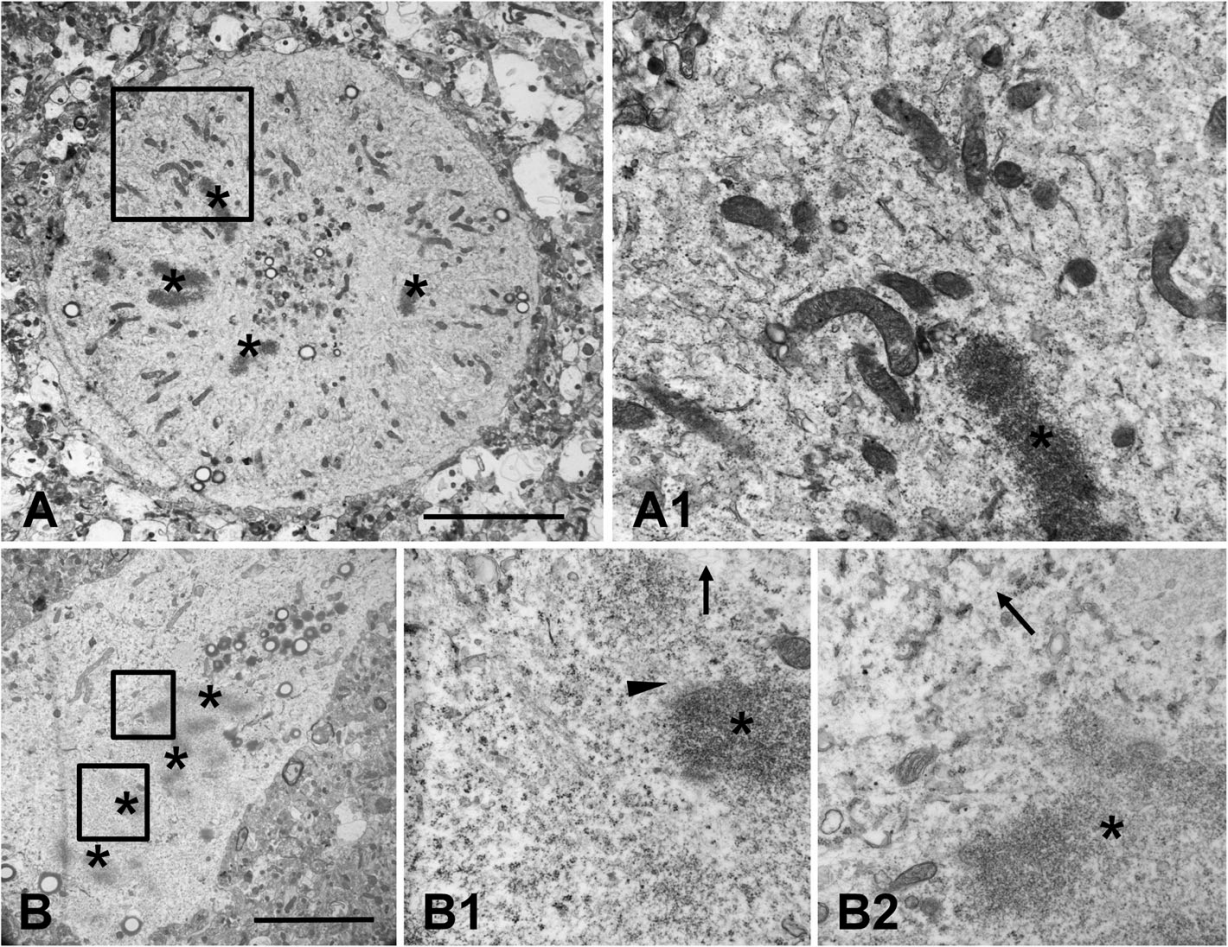
Electron microscopy of astrocytes in arrested mitoses. **a** Condensed chromosomes (asterisks) are distributed over a large volume of this rounded astrocyte cell body. (a1) Intermediate filaments are not found in the cytoplasm near chromosomes. **b** Condensed chromosomes (asterisks) are grouped in one part of the cell body. Microtubules near centromere are indicated with arrowhead (in b1). A few microfilaments (arrows in b1 and b2) are found near chromosomes, intermediate filaments are absent. a1,b1, and b2 – enlarged boxed areas in (**a** and **b**), respectively. Scale bars: a,b = 4 μm

Examination of the ultrastructure of arrested mitoses showed that intermediate filaments were undetectable in the cell body around chromosomes (Fig.10a,b), whereas before mitosis in reactive astrocytes and in multinucleated cells that had passed through arrested mitosis there were many intermediate filaments in the cell bodies (not shown). Based on these observations we concluded that most of the intermediate filaments were disassembled prior to mitosis, likely due to phosphorylation. We used an antibody to phospho-Ser55 vimentin directed to a site that needs to be phosphorylated to effect vimentin depolymerization [9]. This antibody consistently stained the entire cytoplasm of mitotic astrocytes including the cytoplasm between the distributed chromosomes in arrested mitoses (Fig. 3a-c). A commercially available antibody against phosphorylated GFAP did not show reliable staining.

Another protein that accumulates in large amounts is the molecular chaperone, alphaB-crystallin. Immunostaining of astrocytes in abnormal mitoses revealed a diffuse cytoplasmic distribution of immunolabeling and alphaB-crystallin surrounded many of the chromosomes (Fig. 9e).

### Multinuclear astrocytes can survive and subsequently enter into a new cell cycle

**Fig. 11:**
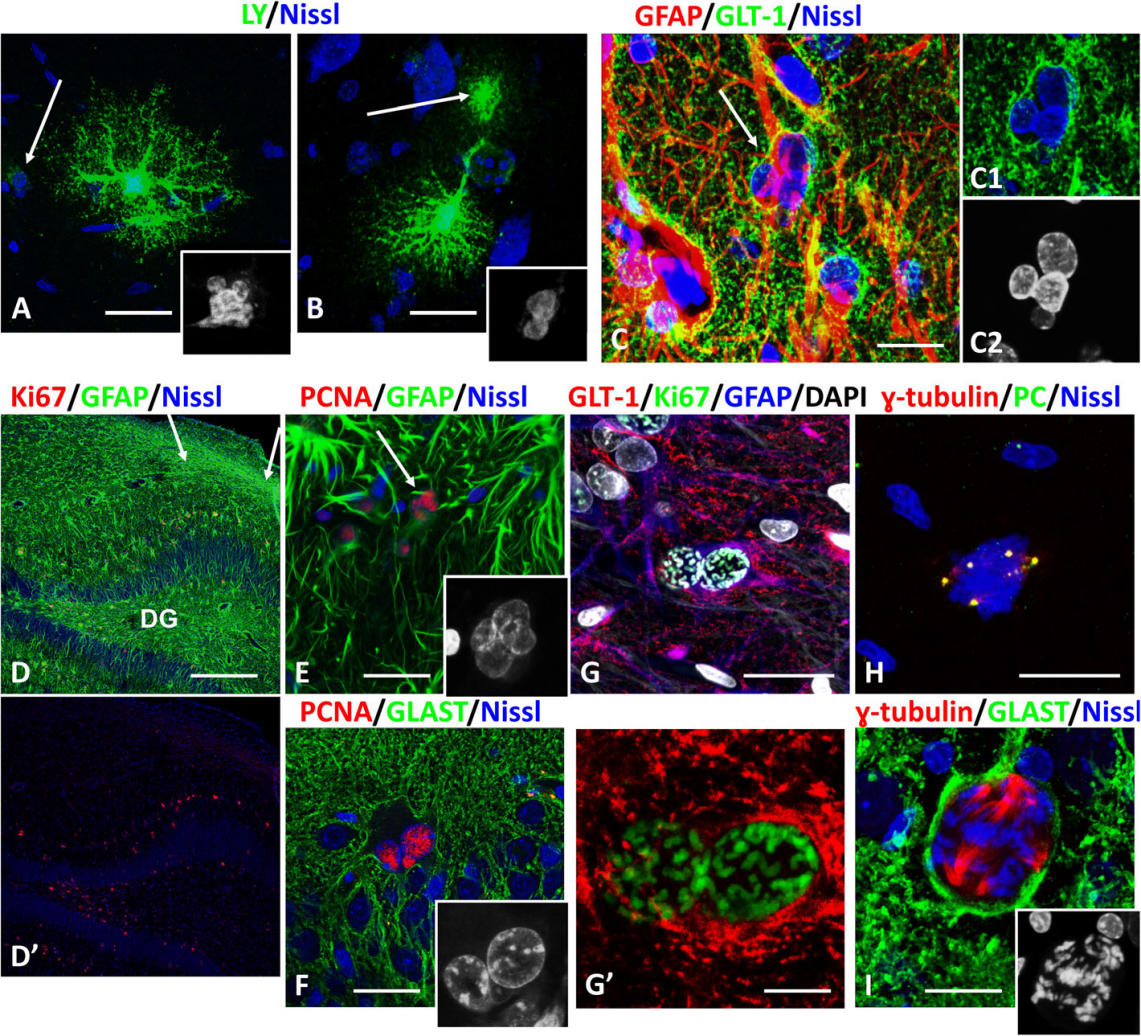
Multinuclear astrocytes can survive for long times and enter new cell cycles. **a**, **b** Multinuclear astrocytes filled with Lucifer Yellow (LY) at 2 (**a**) and 4 (**b**) months after pilocarpine-induced status epilepticus. Note that astrocytes have bushy shape (indicating the presence of many miniature distal processes) and are coupled with neighboring astrocytes (arrows). Insets in lower right parts of images show micronuclei. **c** Gliotic scar in piriform cortex, 1 year after pilocarpine administration. Note multinucleated astrocyte (arrow) with 4 nuclei. GLT-1immunoreactivity outlines the plasma membrane of the cell body. c1 and c2 – enlarged soma (c1) and nuclei visualized with DAPI (c2). **d-i** Multinuclear astrocytes enter new cell cycle and mitosis. Hippocampus, 6 months after pilocarpine administration. **d** Sclerotic hippocampus (arrows indicate the area of neuronal loss and glial scar in the CA1 subfield). Many astrocytes are Ki67+ in dentate gyrus (DG) (d’). **e**, **f** Nuclei in multinuclear astrocytes are PCNA+ indicating that cells are entering the cell cycle. Insets show profiles of several nuclei in each cell. In E the arrow indicates an astrocyte with several PCNA+ nuclei. **g** An astrocyte with two Ki67+ nuclei and condensed chromosomes indicating prophase of mitosis. g’– enlarged image of nuclei in prophase. **h** Metaphase with several centrosomes stained with γ-tubulin and pericentrin (PC). **i** An astrocyte with a large group of chromosomes with several spindle-like structures. Inset shows metaphase chromosomes. All images were obtained with confocal microscope. Scale bars: a,b = 15 μm,  c = 20 μm, d = 90 μm, e,f = 30 μm, g = 20 μm, g',h,i = 8 μm

Reactive astrocytes escaped arrest in metaphase with the formation of many small nuclei. The appearance of multinucleated cells in cell culture has been usually considered as evidence of mitotic catastrophe and cellular demise [7, 51], but recent evidence shows that these cells can survive and enter new cell cycles [25, 39]. In rats after pilocarpine administration, multinucleated astrocytes survived for long periods of time and displayed a morphology similar to normal cells (Fig. 11a, b). The numbers of astrocytes with many (> 5–9 micronuclei, labeled with BrdU) decreased with time, but astrocytes with few (2–3) large nuclei were present in scar tissue even 1 year after the initial insult (Fig. 11c).

We observed several months after pilocarpine-induced seizures that multinucleated astrocytes could enter the cell cycle and divide (Fig. 11d-i). Several observations support this conclusion: 1) immunoreactivity for PCNA appeared in astrocytes with several nuclei (PCNA is mainly expressed during S phase [36] and was never observed in condensed chromosomes and in micronuclei after mitoses) (Fig. 11e,f); 2) in many astrocytes with two Ki67+ nuclei, condensed chromosomes were located at the periphery of the nuclei near the nuclear envelope, a location that is typical for prophase (Fig. 11g); 3) the multiplication of centrosomes in metaphase suggests that two mother centrosomes entered prophase (Fig. 11h); 4) the large numbers of chromosomes outnumbered those in diploid cells (Fig. 11i). Secondary waves of mitotic activity may have been initiated by spontaneous seizures that were consistently present in these animals.

## Discussion

Neuropathologists have recognized “Alzheimer Type 1 astrocytes”/“Creutzfeldt cells” for decades [8, 12], remarking on their hypertrophic size and their multinucleated nature or the abnormal distribution of metaphase chromosomes. Although the appearance of these astrocytes was attributed to abnormal mitoses, a detailed analysis and mechanistic explanation of the changes are lacking. We have examined these cells from a more modern perspective and report several marked pathological findings that underlie the appearance of arrested mitoses. Not only are chromosomes distributed abnormally in mitoses, but also a number of the proteins critical for the normal arrangement of the mitotic spindle are mis-localized. These aberrations are accompanied by severe alterations in cellular shape.

The appearance of Creutzfeldt cells varies in different pathological injuries and between species. For example, in pilocarpine-induced seizures in mice there are fewer of these abnormal astrocytes than in rats (unpublished data). Although we found Creutfeldt astrocytes in all models of injury in the rat brain, pilocarpine seizures produced the largest number. It is difficult to draw comparisons with human CNS injury, but as a general statement, Creutzfeldt astrocytes are described in a number of human neuropathological states but appear to be fewer in number than in the model we have chosen to examine. However, the human Creutzfeldt astrocytes appear to have many similar, even identical, pathological characteristics as the ones we illustrate in our study.

### Hypertrophic astrocytes with arrested mitosis change cell geometry

We observed marked changes in astrocyte shape, including the loss of fine processes, partial retraction of main branches and the rounding up of cell bodies when we examined mitotic, hypertrophic astrocytes. The phenomenon of cell rounding during mitosis has been described and characterized in most detail in cultured cells. Here, typically over a period of 5–20 min, flat cells take on a near-spherical shape [29]. Such shape changes occur due to cooperative interactions between microtubules and a subplasmalemmal actomyosin complex that causes retraction of the cell margins [13, 21].

Astrocytes, in contrast, are process-bearing cells with complex geometry. Astrocytes divide in the young mouse brain without losing miniature peripheral processes ([22]). Similarly, we found reactive astrocytes dividing in the context of pilocarpine seizures that also retained their fine processes (see Fig. 3). However, many of the hypertrophic astrocytes had lost processes. The rounding up of astrocyte cell bodies during mitosis is likely to reflect similar mechanisms seen in the rounding of cultured cells during mitosis. In hypertrophic astrocytes many astral microtubules interact with the plasma membrane along the length of processes that increase the pulling forces to centrosomes and result in their mis-location into proximal processes. Simultaneously the pulling forces of astral microtubules are applied to the plasma membrane that may cause a retraction of astrocyte processes and branches. Whether a loss of miniature processes is typical only for mitosis or may occur at least partially in interphase is not clear.

### Centrosomes are displaced from spindle poles in hypertrophic astrocytes

During a normal mitosis, centrosomes are attached to kinetochore, polar, and astral microtubules, the latter of which attach to the plasma membrane to generate forces applied to centrosomes. However, we found that centrosomes were often displaced away from spindle poles in reactive astrocytes. Although this does not necessarily result in abnormal mitosis, an inability of centrosomes to occupy their correct places may have important, initial roles in the development of mitotic arrest. Displaced centrosomes may not be able to orient spindles properly.

In mitosis in a normal astrocyte, the migration of centrosomes to spindle poles and the subsequent correct orientation of the spindles strongly depends on interactions between astral microtubules that attach to the subplasmalemmal cytoskeleton of the cell body [11, 50]. However, in enlarged astrocytes, many astral microtubules spread into the proximal branches of processes. At this abnormal location, the astral microtubules interact with the plasma membrane to generate pulling forces on the centrosomes. We hypothesize that these alterations have dual effects: 1) more time is needed for astral microtubules to attach to the plasma membrane of proximal astrocytic processes because of the larger space that they must pass through to reach the plasma membrane; and 2) there is an increase in the pulling forces applied to centrosomes (because of the larger surface of plasma membrane), which may cause their displacement from the spindle poles into the proximal parts of processes.

Furthermore, displaced centrosomes may not be correctly connected to kinetochore microtubules that bind centromeres with centrosomes [10, 45]. This may cause the appearance of additional spindle poles without centrosomes and also abnormal interactions between kinetochore microtubules and centromeres. An incorrect attachment of chromosomes to the kinetochore microtubules leads to activation of SAC, which delays progression through mitosis [10, 45].

The phenomenon of multiple or fragmented centrosomes exists in a variety of pathological circumstances. It can follow X-irradiation and is a frequent event in malignancies [33], but neither of these conditions appears appropriate to a discussion of reactive astrocytes. X-irradiation produces DNA damage, and we found no evidence for DNA damage in the reactive astrocytes. We used gamma-tubulin as a marker for centrosomes, and it did localize to the two centrosomes in normal mitoses. Other astrocytes contained many foci of gamma-tubulin, which may represent multiple or fragmented centrioles. However, these foci may or may not contain all of the centriolar machinery. That is, some of these may represent centriolar satellites although such structures, which contain centrin, do not also contain centriolar molecules, including gamma-tubulin [32]. Other molecules include Cep63, which binds to Cdk1 and targets it to the centrosome as a requisite for entry into M phase [33]. In that study, the overexpression of Cep63 or Cdk1 induced centrosome amplification.

The appearance of several spindle pole–like structures during abnormal mitosis indicates that centrosomes, when they are not associated with poles, do not have a leading role in the formation of the mitotic spindle, and that the assembly of kinetochore microtubules is not controlled by centrosomes. In fact, the displacement of centrosomes from spindle poles as well as appearance of several spindle poles may not inevitably leads to arrested mitosis. Abnormal spindles with displaced centrosomes and several pole–like foci of microtubule convergence may be able to perform chromosome segregation after satisfaction of SAC. Thus, an essential role of centrosomes in spindle formation has been reevaluated and may differ in different types of cells [15].

### Many proteins critical for normal mitosis are mis-localized during abnormal mitoses

We found that many of the regulatory components of mitotic organization were mis-localized in the abnormal mitoses. These included gamma-tubulin, NUMA, Aurora A and B kinases, BuBR1 and BUB3. In mitosis, Aurora A associates with TPX2, which targets the kinase to spindle microtubules [27]. In the reactive astrocytes, however, Aurora A is scattered throughout the cytoplasm and many of these Aurora A+ puncta are not associated with TPX2. Thus, the mis-localization of Aurora A may not allow it to be properly associated with spindle microtubules. Since Aurora A needs to be phosphorylated to be activated, a lack of phosphorylation may underlie some of the mis-localization. Aurora A is phosphorylated at Y148 by Src kinase, itself activated by Golgi fragmenting before mitosis. Indeed, Src inhibitors reduce the association of Aurora A with centrosomes [5]. It would be important to examine Aurora A phosphorylation in future studies.

A lack of phosphorylation of Aurora A substrates may also cause mis-localization of critical molecules. For example, one of the substrates of Aurora A kinase is NUMA, and the inactivation of Aurora A causes a loss of NUMA at the cell periphery and an increase at the spindle poles [27]. Another of the Aurora A substrates, centrosomal protein 4.1-associated protein (CPAP), also regulates spindle positioning. Without CPAP phosphorylation, spindles are asymmetric and multipolar and pericentriolar material is not localized, but dispersed [9], in some ways similar to what we observed in the reactive astrocytes. Assessing the phosphorylation states of these critical proteins may shed further light on the mitotic abnormalities.

The roles of Bub3 and BubR1, which have been investigated via knockouts and mutations, principally revolve around promoting the attachment of kinetochores to spindles and organizing chromosomes in normal congression [17]. In the abnormal astrocytes these proteins are mis-localized, presumably either because binding partners such as kinetochore proteins are mis-localized or because they are now bound to other proteins. Thus, the effects of mis-localization of checkpoint kinases may be similar to the effects of knockouts or mutations.

### Hypertrophic astrocytes display a failure of cytokinesis

One of the characteristic pathologies we observed was a failure of cytokinesis, leading to multinucleated cells. This is a highly complex process, involving the formation of a cleavage furrow and then the ingression of the furrow, followed by abscission and separation of the two daughter cells. One of the proteins required for cytokinesis is Aurora B kinase, which is initially localized to the central spindle and then to the furrow along with Mklp1, a kinesin protein, which Aurora B phosphorylates. In v-Src transformed cells, which display cytokinesis failure, both Aurora B and Mklp1 are mis-localized away from the furrow [40, 46]. Multinucleated astrocytes showed abnormal localizations of Aurora B, suggesting that its mis-localization contributes to cytokinesis failure.

In cancer cells, multipolar cell divisions can result in failure of cytokinesis, leading to multinucleated cells [20]. However, in that study, many of the cells that underwent a multipolar division died during mitosis or a following interphase. Other cells ceased cell division. We saw no evidence for cell death in reactive astrocytes, although we could not follow single cells over time. Ganem [20] suggest that multiple centrosomes result in chromosomal instability because they generate chromosomes attached to only one kinetochore (merotely). It is possible that some of the astrocyte chromosomes that appear outside of the spindle may have merotelic attachments.

### Hypertrophic astrocytes contain large amounts of cytosolic proteins that may interfere with mitosis

We speculate that an additional factor that may underlie mis-localization of critical proteins and chromosomes is the large accumulation of cytoplasmic proteins typical for reactive astrocytes, especially the intermediate filament proteins, GFAP, vimentin, and nestin, and the small heat shock protein, alphaB-crystallin. Our observations suggest that the intermediate filaments are depolymerized during mitosis, which is a normal event. But large amounts of cytosolic proteins could mechanically interfere with spindle microtubules and the movement of chromosomes. Indeed, GFAP immunostaining revealed the protein localized throughout the astrocyte cytoplasm, even surrounding chromosomes and appearing in the spindle, localizations from which it is normally excluded during mitosis.

A similar dispersed localization among chromosomes is present for alphaB-crystallin. This small heat shock protein can associate with tubulin and, in vitro, regulate tubulin assembly into microtubules [2, 24]. Specifically, high molar ratios of alphaB-crystallin to tubulin inhibit assembly [24]. Although we do not know molar ratios of these proteins in reactive astrocytes, it is possible that alphaB-crystallin can interfere with microtubule assembly and consequently with several of the critical processes in mitosis. Furthermore, another small heat shock protein, hsp27, also accumulates in reactive astrocytes, and in vitro has tubulin and microtubule binding capacities [23].

High levels of GFAP and small heat shock proteins associated with abnormal chromosome congression and segregation are present in astrocytes of Alexander disease, a severe neurological disorder caused by dominant mutations in *GFAP*, which lead to a massive accumulation of GFAP and small heat shock proteins with the formation of proteinaceous aggregates, Rosenthal fibers. In Alexander disease and its mouse models, many astrocytes display abnormal, C-like mitoses with formation of multinuclear, polyploidal cells and large, multinuclear (2–3 nuclei) astrocytes [33]. These same considerations may help to explain the genesis of abnormal mitoses in hypertrophic astrocytes in a variety of disorders and in hypertrophic glial tumor cells in glioblastomas [3, 14, 48].

### Why do reactive astrocytes undergo abnormal mitosis?

We have used the term “abnormal” mitosis throughout this work, realizing that it does not have a specific meaning, but rather refers to a number of pathological features that include the abnormal positioning of chromosomes in the spindle and a mislocalization of critical mitotic molecules. We have illustrated these abnormalities in several Figures:

**Table:** 

Normal mitosis	Figure 4a
C-type mitosis	Figure 1a and b
Abnormal positioning of chromosomes	Figures 3f, 5m, 6b, f, i, 7g, j, 8d, e, 9a, e, 11i
Abnormal positioning of mitotic molecules	NUMA – Fig. 5o
	Aurora A – Fig. 6f, i
	BUB3, BubR1 – Fig. 7b, c, f, g, j
No exclusion of cytoplasmic proteins from spindles	Figure 9a, e

**Fig. 12:**
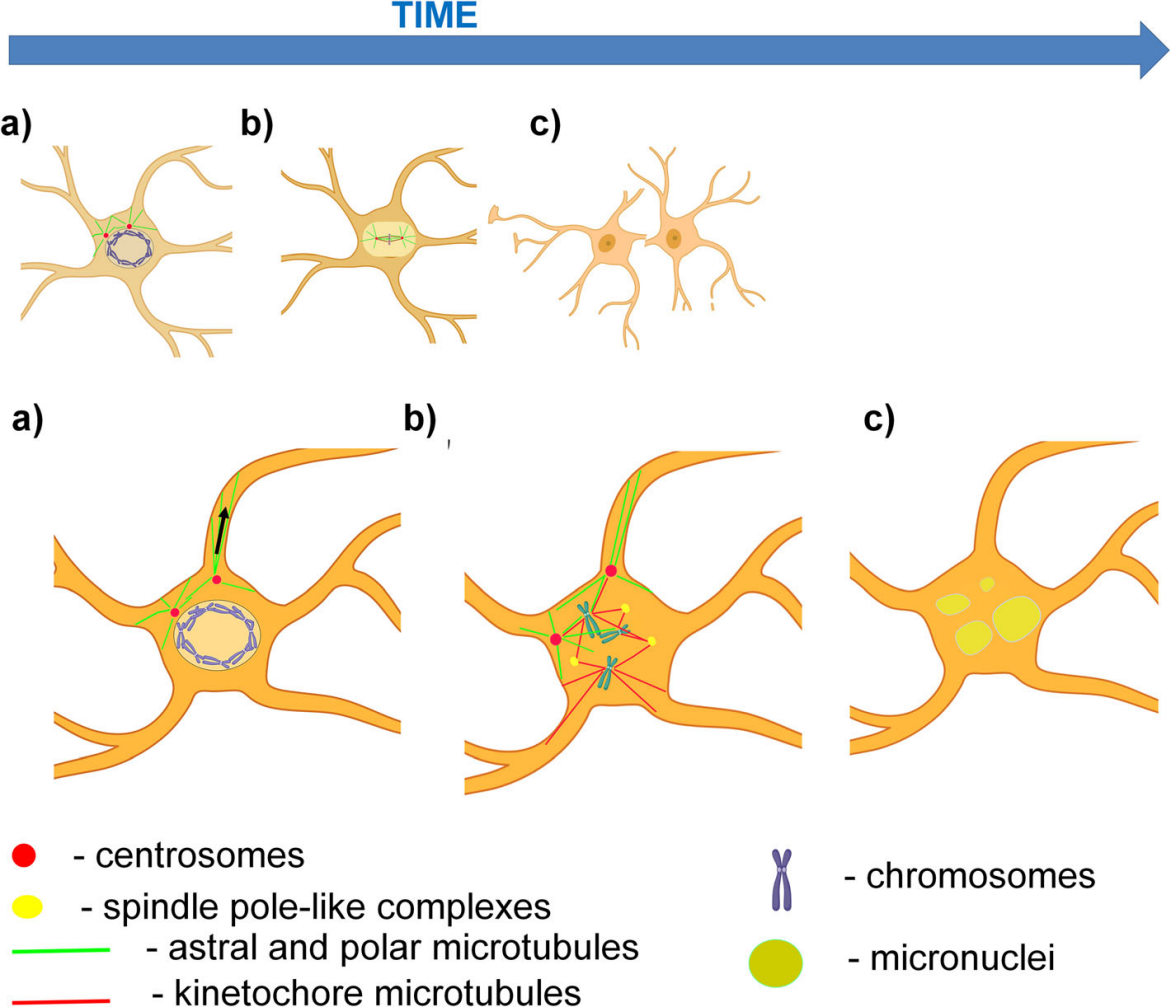
Schematic diagram of arrested mitosis (lower panel) in comparison with normal mitosis (upper panel). **a** Prophase. Due to the enlargement of cell bodies and primary processes in reactive astrocytes, many astral microtubules penetrate into the processes and pull centrosomes away from nuclei (arrow indicates a pulling centrifugal force applied to centrosome). Note that these forces may also pull on the plasma membrane, causing retraction of processes. **b** Metaphase. In arrested mitosis astral, polar and kinetochore microtubules lack a normal arrangement; several pole-like complexes are formed and include NUMA, γ-tubulin, TPX2; chromosomes cannot be congressed at a metaphase plate and consequently correct segregation is not fulfilled. Note, one chromosome is located outside of the metaphase plate in this diagram . Furthermore, in a normal metaphase, soluble proteins, such as GFAP, vimentin, and alphaB-crystallin, are excluded from the spindle area, whereas in the enlarged astrocytes, however, these proteins are not excluded, and intercalate between chromosomes and spindles (note border between lighter area of spindle and darker cytoplasm in the upper Figure, and the absence of such a border in the lower Figure). **c** Isolated or groups of chromosomes are covered with nuclear envelopes with formation of micronuclei

We consider that the main reason for the arrested mitoses is a failure to form mitotic spindles that are able to perform the normal congression of chromosomes into a metaphase plate. This results in an arrest at the prophase - metaphase transition and the consequent mis-distribution of chromosomes into a large volume. Later on, these chromosomes are enclosed by envelopes and give rise to micronuclei. Several factors contribute to these abnormal mitoses. First, the accumulation of proteins that can interfere with microtubule assembly and the interactions of microtubules with substrates will impede spindle formation. Second, the marked changes in the location and distribution of many proteins participating in mitosis (e.g. gamma tubulin, Aurora A, Aurora B, BubR1, BuB3, NUMA) impedes normal mitosis. Third, the complex cellular geometry in which many enlarged processes are unequally distributed along the cell body and the distribution of centrosomes into proximal processes will produce non-symmetrical pulling-pushing forces produced by astral microtubules that interact with the plasma membrane at various distances from the cell body, interfering with the formation of a normal spindle. See Fig. 12 for a diagrammatic illustration of these points.

We found that multinucleated astrocytes could be observed up to a year after an initial excitotoxic insult. While we cannot exclude the possibility that some of the multinucleated astrocytes eventually die, our results support that micronuclei fuse into larger ones, resulting in multinucleated cells with fewer, larger nuclei over time [14]. Nuclear fusion occurs in cell culture [49] and in some synkaryons [41].

## Conclusions

1) Abnormal mitoses are common in proliferating astrocytes reacting to a variety of brain insults, but possible mechanisms underlying this pathology have remained unknown.

2) Arrested mitoses appear due to abnormal formation of mitotic spindles.

3) Several factors predispose to the formation of abnormal spindles, including changes in cell geometry and the accumulation of large amounts of cytoplasmic proteins.

4) Multinucleated astrocytes can survive long periods of time and even re-enter mitosis.

## Supplementary information

**Additional file 1.**

**Additional file 2.**

**Additional file 3.**

**Additional file 4.**

**Additional file 5.**

## Data Availability

Not applicable.
